# The impact of physical frailty on the response to inactivated influenza vaccine in older adults

**DOI:** 10.18632/aging.202207

**Published:** 2020-12-09

**Authors:** Krissy K. Moehling, Bo Zhai, William E. Schwarzmann, Uma R. Chandran, Marianna Ortiz, Mary Patricia Nowalk, David Nace, Chyongchiou J. Lin, Michael Susick, Min Z. Levine, John F. Alcorn, Richard K. Zimmerman

**Affiliations:** 1 Department of Family Medicine, University of Pittsburgh, Pittsburgh, PA 15260, USA; 2 Department of Pediatrics, University of Pittsburgh, Pittsburgh, PA 15213, USA; 3 Division of Pulmonary Medicine, Department of Pediatrics, University of Pittsburgh, Pittsburgh, PA 15224, USA; 4 Department of Biomedical Informatics, University of Pittsburgh, Pittsburgh, PA 15261, USA; 5 Division of Geriatric Medicine, University of Pittsburgh, Pittsburgh, PA 15261, USA; 6 Ohio State University College of Nursing, Columbus, OH 43210, USA; 7 National Center for Immunization and Respiratory Diseases, Influenza Division, Centers for Disease Control and Prevention, Atlanta, GA 30329, USA

**Keywords:** frailty, immune response, antibody titers, peripheral cell mediated immunity, influenza

## Abstract

Physical frailty’s impact on hemagglutination inhibition antibody titers (HAI) and peripheral blood mononuclear cell (PBMC) transcriptional responses after influenza vaccination is unclear. Physical frailty was assessed using the 5-item Fried frailty phenotype in 168 community- and assisted-living adults ≥55 years of age during an observational study. Blood was drawn before, 3, 7, and 28 days post-vaccination with the 2017-2018 inactivated influenza vaccine. HAI response to the A/H1N1 strain was measured at Days 0 and 28 using seropositivity, seroconversion, log_2_ HAI titers, and fold-rise in log_2_ HAI titers. RNA sequencing of PBMCs from Days 0, 3 and 7 was measured in 28 participants and compared using pathway analyses. Frailty was not significantly associated with any HAI outcome in multivariable models. Compared with non-frail participants, frail participants expressed decreased cell proliferation, metabolism, antibody production, and interferon signaling genes. Conversely, frail participants showed elevated gene expression in IL-8 signaling, T-cell exhaustion, and oxidative stress pathways compared with non-frail participants. These results suggest that reduced effectiveness of influenza vaccine among older, frail individuals may be attributed to immunosenescence-related changes in PBMCs that are not reflected in antibody levels.

## INTRODUCTION

The burden of influenza-related morbidity and mortality among older adults is substantial. Surveillance studies estimate that 71%-85% of influenza deaths occur in adults ≥65 years of age [1]. Adults ≥65 years are 10 to 30 times more likely than younger adults to experience acute respiratory failure attributed to influenza disease [2]. Other complications include higher rates of pneumonia, stroke and heart attack [3]. Low vaccine effectiveness among the elderly has been attributed to senescence of the immune system and a decreased immune response to vaccine antigens. Decreased humoral and cell-mediated immune responses have been reported specific to influenza vaccination in older adults compared with younger adults [4], including altered T-cell function and overall declines in cell-mediated adaptive immunity [3].

Overlaid on age-related immunosenescence are the effects of frailty [4], a multi-dimensional syndrome marked by losses in function and physiological reserve [5]. Physical frailty, characterized by diminished strength, endurance, and reduced physiologic function [6], leads to increased risk of acute illness, falls, disability, hospitalization, institutionalization and mortality [5, 7].

Frailty’s impact upon older adults’ antibody immune response outcomes after influenza vaccination is unclear. A 2011 study found that physical frailty among community-dwelling adults >70 years of age was associated with diminished immunological response to influenza vaccine and more severe influenza-like illness [8]. More recent studies have found no difference in post-vaccination antibody titers between frail and non-frail or between frail and pre-frail community-dwelling older adults ≥65 years [9–12].

Efforts to develop more effective vaccines have driven research on humoral and cell-mediated immune responses to influenza virus. These studies [13–16] have focused on general subpopulations of older adults without consideration of factors that may directly affect the degree of immunosenescence, such as physical frailty. The purpose of this study was to assess hemagglutination inhibition antibody (HAI) titers and peripheral blood mononuclear cell (PBMC) transcriptomic responses to influenza vaccine in the context of physical frailty status.

## RESULTS

### Demographics

Characteristics for the entire cohort as well as by frailty status among the entire cohort and PBMC subgroup are presented in Table 1. Overall, participants were primarily female (68.5%) and white (76.2%). Forty-seven percent were taking a statin medication, 32.5% noted having ≥2 chronic health conditions, and 41.1% were obese. Median age was 71.5 years (64.9-83.1). Overall, frailty was low, with 34.5% of the cohort considered frail; frail persons had 2-4 “frailty components.” Frail individuals were significantly older (74.3 vs. 70.5 years; *P* = 0.040), were more likely to have 1 or more high risk condition (*P* = 0.007) and had lower ADL (13.0 vs. 14.0; *P* < 0.001) and IADL (13.0 vs. 14.0; *P* < 0.001) scores. Frail individuals in the PBMC subgroup were significantly older (82.9 vs. 67.5 years, *P* = 0.012), had higher BMI (31.0 vs. 26.4, *P* = 0.045), and as expected, scored lower on functional status measures (13.0 vs.14.0, *P* = 0.002 for ADL and 11.0 vs.14.0, *P* = 0.009 for IADL).

**Table 1:** Demographics on entire cohort and subset analytic group by frailty status.

**Variables**	**Entire cohort (N=168)**	**Frail (N=58)**	**Non-frail (N=110)**	***P* value^1^**	**PBMC subset Frail (N=13)**	**PBMC subset Non-frail (N=15)**	***P* value^1^**
Age, yr, Median (Q1, Q3)	71.5 (64.9,83.1)	74.3 (66.4,88.2)	70.5 (63.3,81.0)	**0.040**	82.9 (72.7,88.1)	67.5 (62.9,72.9)	**0.012**
Female sex, N (%)	115 (68.5)	41 (70.7)	74 (67.3)	0.651	9 (69.2)	12 (80.0)	0.670
Caucasian race, N (%)	128 (76.2)	46 (79.3)	82 (74.6)	0.491	12 (92.3)	14 (93.3)	1.000
Non-Hispanic, N (%)	165 (98.2)	58 (100.0)	107 (97.3)	0.552	13 (100.0)	14 (93.3)	1.000
BMI, Median (Q1, Q3)	27.8 (24.5,33.1)	29.7 (24.4,35.1)	27.4 (24.5,31.1)	0.075	31.0 (29.3,34.8)	26.5 (24.7,30.5)	**0.045**
Current smoker, N (%)	22 (13.1)	10 (17.2)	12 (10.9)	0.247	1 (7.7)	1 (6.7)	1.000
1 high-risk condition,^2^ N (%), ref. = 0	59 (36.2)	22 (37.9)	37 (35.2)	**0.007**	5 (38.5)	4 (28.6)	0.555
≥ 2 high-risk conditions^2^, N (%), ref. = 0	53 (32.5)	26 (44.8)	27 (25.7)		6 (46.2)	5 (35.7)	
Current statin medication use, N (%)	79 (47.0)	31 (53.5)	48 (43.6)	0.226	8 (61.5)	5 (33.3)	0.255
ADL score, median (Q1, Q3)^3^	14.0 (13.0,14.0)	13.0 (13.0,14.0)	14.0 (14.0,14.0)	**<0.001**	13.0 (13.0,13.0)	14.0 (14.0,14.0)	**0.002**
IADL score, median (Q1, Q3)^3^	14.0 (13.0,14.0)	13.0 (8.0,14.0)	14.0 (13.0,14.0)	**<0.001**	11.0 (7.0,14.0)	14.0 (13.0,14.0)	**0.009**
0-1 Frailty components (non-frail), N (%)	110 (65.5)	-	-	-	-	-	-
≥ 2 Frailty components (frail), N (%)	58 (34.5)	-	-	-	-	-	

^1^Chi-square/Fisher’s Exact for categorical variables, Wilcoxon for continuous variables.

^2^Comorbidities include: diabetes, heart disease, asthma, chronic lung disease, blood disorders, kidney disorders, liver disease, neurological disorders, osteoporosis.

^3^ADL and IADL, scores range from 0-14, higher scores indicate greater functionality.

### HAI results

Pre- and post-vaccination A/H1N1 HAI antibody titers of the entire cohort and for the PBMC subgroup by frailty status are reported in Table 2. Nearly half (47.6%) of the cohort was considered seropositive at Day 0 rising to 80.3% seropositivity at Day 28. Only 35.7% of the cohort seroconverted 28 days post-vaccination, with a mean fold-rise in the log_2_ titer ratio of 1.44 ± 0.58 for the cohort. There were no significant differences between frailty subgroups in any HAI response outcome.

**Table 2:** Pre- and post-vaccination A/H1N1/Michigan/45/2015-pdm09-like virus antibody titers.

**HAI response to A/H1N1**	**Entire cohort (N=168)**	**Frail (N=58)**	Non-frail* (N=110)	**PBMC subset**	
				**Frail (N=13)**	**Non-frail^1^ (N=15)**
Day 0 log_2_ HAI titer, Mean ± SD	4.86 ± 1.87	4.70 ± 1.96	4.94 ± 1.83	3.78 ± 1.66	4.19 ± 1.81
Day 28 log_2_ HAI titer, Mean ± SD	6.31 ± 1.69	6.30 ± 1.75	6.32 ± 1.67	5.82 ± 1.34	5.89 ± 1.27
Day 0 seropositivity rate, N (%)	80 (47.6)	24 (41.4)	56 (50.9)	3 (23.1)	5 (33.3)
Day 28 seropositivity rate, N (%)	135 (80.3)	45 (77.6)	90 (81.8)	8 (61.5)	12 (80.0)
Day 28 seroconversion rate, N (%)	60 (35.7)	21 (36.2)	39 (35.5)	5 (38.5)	6 (40.0)
Day 28 fold-rise in log_2_ HAI titer, Mean ± SD	1.44 ± 0.58	1.50 ± 0.64	1.41 ± 0.55	1.77 ± 0.81	1.59 ± 0.58

Seropositivity = HAI titer ≥40; Seroconversion (4-fold rise in post-vaccination titer at Day 28 given Day 0 titer ≥10).

^1^All *P* values for tests >0.05 for differences between frail and non-frail groups; Chi-square/Fisher’s Exact for categorical variables; t-test for continuous variables.

Multivariable regression was performed on data from the entire cohort to determine predictors of H1N1 antibody response. Frailty was not significantly associated with any Day 28 measure of HAI titers when adjusting for demographic factors. Day 0 log_2_ HAI titer, age, and sex were significantly associated with seroprotection and seroconversion (Table 3). Younger age and being female were significantly related to higher Day 28 seropositivity and seroconversion.

**Table 3:** Predictors of influenza A/H1N1/Michigan/45/2015-pdm09-like virus antibody titers from multivariable regression for entire cohort (N=168).

**Outcomes from logistic regression**	**OR (95% CI)**	***P* value**
*A/H1N1*^1^ *Day 28 Seropositivity (HAI titer ≥ 1:40)*		
Frail, ref.=non-frail	1.16 (0.45-2.97)	0.754
Age, years	0.95 (0.91-0.99)	**0.008**
Female, ref.=male	2.84 (1.07-7.51)	**0.036**
A/H1N1 log_2_ Day 0 HAI titers	2.12 (1.54-2.92)	**<0.001**
*A/H1N1 Day 28 Seroconversion (4-fold rise)*		
Frail, ref.=non-frail	1.17 (0.54-2.54)	0.698
Age, years	0.94 (0.91-0.98)	**0.002**
Female, ref.=male	2.76 (1.19-6.43)	**0.019**
A/H1N1 log_2_ Day 0 HAI titers	0.59 (0.47-0.74)	**<0.001**
**Outcomes from linear regression**	**Beta (SE)**	***P* value**
*A/H1N1 Log_2_ Day 28 HAI titers*		
Frail, ref.=non-frail	0.27 (0.22)	0.233
Age, years	-0.03 (0.01)	0.001
A/H1N1 log_2_ Day 0 HAI titers	0.51 (0.06)	<0.001
*A/H1N1 Log_2_ fold-rise in HAI titers (Day 28/Day 0)*		
Frail, ref.=non-frail	0.05 (0.07)	0.492
A/H1N1 log_2_ Day 0 HAI titers	-0.20 (0.02)	<0.001

^1^A/H1N1=A/H1N1/Michigan/45/2015-pdm09-like virus.

### PBMC results - differentially expressed genes (DEG) between frail and non-frail groups at Days 0, 3 and 7

Table 4 shows the DEGs that were significantly different between frail and non-frail groups at Days 0, 3 and 7. In total, there were 584, 597, and 776 DEGs at Day 0, 3, and 7, respectively. At all three time points, frail adults showed lower expression of genes in interferon signaling pathways, suggesting lower baseline interferon activation compared to the non-frail group. Several interferon-induced proteins, *IFI6, IFIT1, IFIT3, IFITM3, MX1*, and *ISG15* were decreased in the frail adults. Of note, interferon-inducible transmembrane-1 (*IFIT1*), MX dynamin-like GTPase 1 (*MX1*), and interferon stimulated gene (*ISG15*) were down regulated > 1 log ratio at all three time points (Supplementary Table 1). Other pathways involved in vaccine responses, including activation of interferon regulatory factor, (*IRF*) by cytosolic pattern recognition receptors on Day 0, communication between innate and adaptive immune cells on Day 3, and B cell receptor signaling on Day 0 and Day 7, were decreased in the frail adults, suggesting reduced basal activation of both innate and adaptive immune responses.

**Table 4:** Differentially expressed pathways between frail (N=13) and non-frail (N=15) individuals on Day 0 (pre-vaccination) and Days 3 and 7 post-vaccination analyzed by Ingenuity pathways analysis.

	**Day 0 pre-vaccination**		**Day 3 post-vaccination**		**Day 7 post-vaccination**	
**Ingenuity canonical pathways**	**Upregulated genes**	**Downregulated genes**	**Upregulated genes**	**Downregulated genes**	**Upregulated genes**	**Downregulated genes**
Activation of IRF by cytosolic pattern recognition receptors	--	ADAR, DHX58, IFIT2, IRF7, IRF9, ISG15, STAT2, ZBP1	--	--	IFNAR1	DHX58, IFIT2, IKBKE, IRF7, ISG15, STAT2, ZBP1
B cell receptor signaling	RAP2A	FOXO1, IGHG2, IGHG3, IGHG4, PIK3CD, PRKCB, PTK2, RASSF5	--	--	KRAS, NFAT5, PPP3CA, PPP3CB, PPP3R1, PAP2A	ETS1, IGHA1, IGHG1, IGHG2, IGHG3, IKBKE, MAPK11
Communication between innate and adaptive immune cells	--	--	--	CCR7, CD4, CD40LG, IGHA1, IGHG2, IGHG3, TNFRSF12B	--	--
EIF2 signaling	--	--	--	--	EIF1, KRAS, PPP1CB, RAP2A, RPL36A	CCND1, EIF4A3, RPL10A, RPL14, RPL18, RPL23A, RPL26, RPL31, RPL32, RPL34, RPL36, RPL37, RPL37A, RPS13, RPS19, RPS20, RPS23, RPS24, RPS25, RPS27A, RPS28, RPS4X, RPS6, RPS8,
Glutathione biosynthesis	GCLC	--	GCLC, GCLM	--	GCLC, GCLM	--
GM-CSF signaling	--	--	--	--	KRAS, PPP3CA, PPP3CB, PPP3R1, RAP2A	CCND1, ETS1
Heme biosynthesis II	CPOX, HMBS, UROS	--	--	--	--	--
HIF 1-alpha signaling	ELOB, MMP8, PDGFC, RAP2A, RBX1, SLC2A5	EGLN3, HIF1A, NOS3, PIK3CD	--	--	--	--
IL-8 signaling	AZU1, DEFA1, GNA12, GNG10, GPLD1, PDGFC, RAP2A, RHOU	CCND1, CXCR2, ITGAX, PIK3CD, PRKCB, PTK2	AZU1, DEFA1, GNA12, MAPK9, MPO, RAB11FIP2, RAP2A, TEK	CCND1, PRKCA, PTK2	--	--
Interferon signaling	--	IFI6, IFIT1, IFIT3, IFITM3, IRF9, ISG15, MX1, OAS1, STAT2	--	IFI35, IFIT1, IFITM3, ISG15, MX1, OAS1, STAT2	IFNAR1	BAK1, IFI6, IFIT1, ISG15, MX1, OAS1, STAT2
Regulation of IL-2 expression in activated and anergic T lymphocytes	--	--	--	--	KRAS, NFAT5, PPP3CA, PPP3CB, PPP3R1, PAP2A, TGFBR1	IKBKE
T cell exhaustion signaling pathway	PDCD1LG2, PPP2R5B, RAP2A	FOXO1, HLA-E, IL6R, IRF9, LGALS9, PIK3CD, STAT2	--	--	BMPR2, IFNAR1, KRAS, NFAT5, PPP2R5B, PPP2R5E, PRDM1, RAP2A, TGFBR1	CTLA4, HLA-G, LGALS9, STAT2

-- Pathway not significantly differentially expressed or no DEGs in category.

*IL-8* signaling was increased in the frail adults on Day 0 and Day 3, corroborating previous studies that increased *IL-8* expression occurs in frailty and immunosenescence. Additionally, on Day 7 post-vaccination, several nuclear factors and signal transduction proteins shared by two pathways, regulation of *IL-2* expression in activated and anergic T lymphocytes and *GM-CSF* signaling were upregulated in the frail group. These results suggest differential activation of the immune responses to vaccination in frail compared to non-frail adults.

On Day 7, eukaryotic initiation factor 2 (*EIF-2*) pathway showed reduced expression in the frail group compared to non-frail suggesting that a decreased protein production response may contribute to reduced immune response to influenza vaccination. This finding may be relevant, as antibody response has long been recognized as the primary aspect of immunogenicity by influenza vaccines [17, 18].

Cyclin D1 (*CCND1*) expression showed consistently lower expression in the frail group at all three time points, at log ratios of -1.7, -1.82, and -1.83, respectively (Supplementary Table 1). These results show reduced cell proliferation in PBMCs in frail adults at Day 0, suggesting senescence in PBMC populations. Moreover, the T cell exhaustion pathway was upregulated in the frail adults on Day 0 and 7 post-vaccination. Both phenotypes are associated with immunosenescence [19].

Pathways involved in oxidative stress were upregulated in the frail group, including *HIF-1α* signaling, HEME biosynthesis II on Day 0, and glutathione biosynthesis on all days. Additionally, the histamine biosynthesis pathway expression, represented by histidine decarboxylase, was decreased at all three time points, at log ratios -1.44, -1.58, and -1.59 (Supplementary Table 1) on Day 0, Day 3, and Day 7, respectively. These results suggest increased oxidative stress and an alternative inflammatory state in the frail group’s PBMC populations.

### Changes in DEG between Days 0 and 3 and between Days 0 and 7 in frail and non-frail groups

Vaccine-induced differences in gene expression between baseline (Day 0) and after vaccination (Day 3 or 7) were assessed. Table 5 shows significant differences in DEGs between Days 0 and 3 and between Days 0 and 7 in frail and non-frail groups. Relatively few genes were differentially expressed at 3 versus 7 days following vaccination (386 frail, 265 non-frail, Supplementary Table 2). However, the DEGs from Day 0 to either Day 3 or Day 7 differed by frailty status. Vaccination in frail patients altered gene expression in T and B cell signaling pathways and crosstalk between dendritic cells and natural killer cells. In addition, DEGs were observed in stem cell pathways. In non-frail patients DEGs were identified in oxidative stress pathways and adaptive immune activation.

**Table 5:** Differentially expressed pathways between Day 0 (pre-vaccination) and Day 3 post-vaccination among frail (N=13) and non-frail individuals (N=15), analyzed by Ingenuity pathways analysis.

	**Frail Day 0 – Day 3**		**Non-frail Day 0 – Day 3**	
**Ingenuity canonical pathways**	**Upregulated genes**	**Downregulated genes**	**Upregulated genes**	**Downregulated genes**
Altered T cell and B cell signaling in rheumatoid arthritis	FASLG	SPP1, TNFRSF13B, TNFRSF13C	--	--
B cell development	--	--	--	CD19, IGHD
Communication between innate and adaptive immune cells	--	--	CCL3L3, HLAG	IGHD
Crosstalk between dendritic cells and natural killer cells	FASLG, ITGAL, KIR3DL1, PRF1	--	--	--
Gluathione redox reactions I	--	--	GSTM1, MGST2	--
Human embryonic stem cell pluripotency	BMP8B, MRAS, PDGFRB, S1PR5, SMAD7	WN10A, WNT16	--	--
NAD biosynthesis II	--	--	IDO1, NMNAT3	--
Transcriptional regulatory network in embryonic stem cells	EOMES, H4C11	H4C14	--	--

-- Pathway not significantly differentially expressed or no DEGs in category.

Table 6 shows significant differences in DEGs between Days 0 and 7 in frail and non-frail groups (944 frail, 1252 non-frail, Supplementary Table 2). In the frail adults, genes and pathways involved in protein production (*EIF2*) and energy consumption (oxidative phosphorylation) were down regulated on Day 7, compared to Day 0. In contrast, in the non-frail group these genes and pathways were upregulated. These results suggest that frailty is correlated with reduced immune cell protein production and energy generation/consumption. In addition, induction of antigen presentation pathway genes was only observed in the non-frail group.

**Table 6:** Differentially expressed pathways between Day 0 (pre-vaccination) and Day 7 post-vaccination among frail (N=13) and non-frail individuals (N=15), analyzed by Ingenuity pathways analysis.

	**Frail Day 0 - Day 7**		**Non-frail Day 0 - Day 7**	
**Ingenuity canonical pathways**	**Upregulated genes**	**Downregulated genes**	**Upregulated genes**	**Downregulated genes**
Antigen presentation pathway	--	--	HLA-DPA1, HLA-DPB1, HLADQA1, HLA-DQB2, HLA-DRA, HLA-DRB1, HLA-DRB5, HLA-G, PSMB6, PSMB8	--
EIF2 signaling	IGF1R	EIF3C, RPL10, RPL11, RPL13A, RPL15, RPL22L1, RPL23, RPL26, RPL27, RPL29, RPL3, RPL31, RPL34, RPL35, RPL37, RPL39, RPL4, RPL5, RPL6, RPL7, RPL7A, RPL9, RPL10, RPS13, RPS14, RPS15A, RPS17, RPS18, RPS19, RPS20, RPS21, RPS23, RPS24, RPS27, RPS27A, RPS29, RPS3A, RPS4Y1, RPS5, RPS6, RPS7, RPS8, RPS9, RPSA	AFT3, EIFAY, EIF2S2, EIF3D, EIF3E, EIF3I, EIF3K, EIF3L, EIF4A3, MT-RNR2, PAIP1, RPL10, RPL10A, RPL12, RPL13, RPL14, RPL18, RPL19, ROL21, RPL22, RPL22L1, RPL23, RPL23A, RPL24, RPL26, RPL27A, RPL29, RPL30, RPL31, RPL32, RPL34, RPL35, RPL35A, RPL36, RPL36A, RPL37, RPL37A, RPL38, RPL39, RPL4, RPL5, RPL6, RPL7, RPL7A, RPLP2, RPS10, RPS11, RPS12, RPS13, RPS14, RPS15, RPS19, RPS2, RPS20, RPS21, RPS23, RPS25, RPS26, RPS27A, RPS3, RPS3A, RPS4X, RPS5, RPS7, RPS8, RPSA, TRIB3, UBA52	AGO4, GSK3B, PIK3CG, PIK3R6
NRF2-mediated oxidative stress response	--	--	AKR7A2, CDC34, DNAJA1, DNAJB1, DNAJC8, FTL, GSTO1, JUND, MGST2, PPIB, PRDX1, SOD1, UBB	DNAJ5, GSK3B, MAP2K5, NQO2, PIK3CG, PIK3R6, PRKCE
Oxidative phosphorylation	MT-CYB, MT-ND1, MT-ND2, MT-ND5	ATP5ME, ATP5PB, COXA2, COX7B, NDUFA6, NDUFS3, UQCRQ	ATP5F1A, ATP5F1B, ATP5F1E, ATP5MC1, ATP5ME, ATP5MF, ATP5MG, ATP5PB, ATP5PD, ATP5PO, COX4I1, COX5A, COX5B, COX6A1, COX6B1, COX7A2, COX7C, COX8A, CYC1, NDUFA1, NDUFA12, NDUFA2, NDUFA3, NDUFA4, NDUFA8, NDUFB10, NDUFB2, NDUFB3, NDUFB7, NDUFB9, NDUFS3, NDUFS5, NDUFS6, UQCR10, UQCR11, UQCRB, UQCRH, UQCRQ	--

-- Pathway not significantly differentially expressed or no DEGs in category.

In the frail group, 66 ribosomal protein genes were down regulated on Day 7. At the same time in the non-frail group, 80 ribosomal protein genes were upregulated on Day 7 (Supplementary Table 3). The *EIF-2* pathway was down regulated post-vaccination in the frail group on Day 7 compared to Day 0, while in the non-frail group, *EIF-2* was up-regulated, suggesting increased protein synthesis in the non-frail group.

In the frail group, six immunoglobulin genes were down regulated on Day 7 compared to Day 0. In contrast, in the non-frail adults, 19 immunoglobulin genes were upregulated in the same comparison (Supplementary Table 4). These findings suggest reduced antibody production, although HAI did not differ between groups. Decreased antibody production is thought to be a major aspect of decreased immune responses in the elderly, and frail adults [20].

In the frail adults, three human leukocyte antigen (*HLA*) genes were found to be down regulated on Day 7 compared to Day 0. By comparison, 8 *MHCII* genes were upregulated in the same comparison in non-frail adults, which is expected as part of the immune responses to vaccines (Supplementary Table 5).

**Figure 1:**
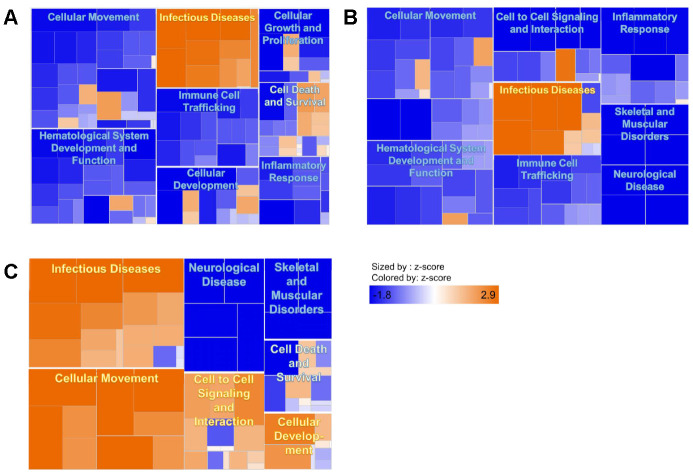
****Heat maps comparing frail to non-frail on day 0 (**A**), day 3 (**B**), and day 7 (**C**) post-vaccination. Lower expression of genes involved in cellular movement, hematological development and function, immune cell trafficking, and inflammatory response can be observed in the frail group compared with non-frail on day 0 and day 3. Higher expression of genes involved cellular movement, cell-to-cell signaling and interaction, and cellular development is shown in the frail group compared with non-frail on day 7. Genes involved in infectious diseases were consistently expressed at higher levels in frail versus non-frail adults at all three time points.

For an overall view of the differences between frail and non-frail groups in DEGs, three heat maps (Day 0, Day 3 and Day 7) were developed. These immune pathway heat maps show that at Day 0, genes involved in cellular movement, hematological system development and function, immune cell trafficking, cellular development, cellular growth and proliferation, and inflammatory response displayed reduced expression in the frail group compared to non-frail (Figure 1A). A similar pattern of reduced immune response genes in the frail adults can be observed on Day 3 (Figure 1B). On Day 7, genes involved in cellular movement and cellular development showed increased activities in the frail adults compared to non-frail (Figure 1C), suggesting delayed immune responses to influenza vaccination. In the frail adults, at all three time points, several gene annotations for viral infectious diseases, including replication of herpesviridae, replication of RNA virus, and replication of viral replicon, showed increased activities, presented by positive activation z-scores (>2) (Supplementary Table 6), suggesting that pre-existing infections may be more prevalent in this group.

## DISCUSSION

Immunosenescence, chronically high levels of circulating inflammatory cytokines (inflammaging), and the overall declines evident in both innate and adaptive immunity that come with aging reduce vaccination responses in older adults [21, 22]. Other factors associated with aging may also reduce the effectiveness of vaccines among older adults. Physical frailty a common, but not universal correlate of advancing age, [23, 10] is one such factor that may further attenuate immune response to vaccines. Recent studies have not demonstrated significant differences in post-influenza vaccination antibody response between frail and non-frail adults ≥65 years [9–12]. The current study confirms previous research; however, significant differences in PBMCs were evident between frail and non-frail groups suggesting differences in the degree of cell-mediated immunity (CMI) activation.

We found many differences in gene expression of PBMCs between the frail and non-frail groups. The T cell exhaustion pathway in frail adults was upregulated on Day 0 and 7 post-vaccination compared to the non-frail adults. T cell exhaustion, particularly programmed cell death protein 1 (*PD1*) and its ligands *PDL1/2* signaling have been widely studied in tumor immunotherapies [24]. Recent studies have shown that increased *PD1* expression was associated with more severe influenza infection [25], and blockade of the *PD1/PDL1* axis enhanced influenza viral clearance in mice [26]. Trivalent inactivated vaccine did not induce immune-related adverse events in cancer patients receiving *PD-1* therapies [27, 28], and *PD-1* treatment was associated with rapid increase of seral conversion among cancer patients, when compared with healthy controls, and cancer patients undergoing cytotoxic chemotherapy [28]. Our results suggest that T cell exhaustion after influenza vaccination is a phenotype of adult frailty. *PD-1* blockade therapy aiming to reduce T cell exhaustion may prove effective in boosting vaccine responses in frail adults.

Expression of several interferon-inducible anti-viral genes including *IFIT1* [29–31], interferon-inducible human oligoadenylate synthetase family (*OAS*) proteins *OAS1, OAS2*, and *OAS-like* protein [32, 33], viperin [34], interferon stimulated exonuclease gene 20 (*ISG20*) [35], and MX1 [36] were expressed at consistently lower levels in frail adults. Competent interferon signaling has been recognized as a critical response to influenza vaccines [37, 38] and to mount an effective host defense against influenza infection [39, 40]. The impact of aging and frailty on interferon signaling is unclear. *IFIT1, IFIT3* [41], *IFITM3* [29], *MX1* [42], and *ISG15* [43] have all been shown to possess anti-influenza activities; however, a genetic variant of *IFITM3* has been associated with severe influenza infection and higher hospitalization rate [30, 44]. Wild type mice treated with exogenous interferon had a higher survival rate and lower viral titers compared to *MX1^-/-^* mice after influenza A/H1N1 and A/H5N1 infections [42, 45]. Another study found *MX1* was protective against influenza A/H1N1 in mice and discovered reduced monocyte interferon-β expression in aged human subjects [46]. We observed reduced interferon signaling in frail adults at Days 0, 3, and 7 post-vaccination. Our results suggest that increasing type-1 interferon signaling may provide therapeutic targets for influenza treatment in the elderly, and for developing influenza vaccine adjuvants.

Previous studies have suggested repeated cytomegalovirus (CMV) and Epstein–Barr virus (EBV) infections, both of which are in the family of herpesviridae, may contribute to immunosenescence and reduced immunogenicity of influenza vaccination [47–50]. Chronic infection with CMV and EB virus have been associated with frailty, and have been recognized as potential factors causing immunosenescense [47, 49, 51]. CMV in particular causes “memory inflation” in CD8+ cells, meaning repeated infection with different types of CMV causes expansion of antigen-specific CD8+ cells reactive to specific CMV strains’ proteins. These CMV-specific populations can take up as much as 50% of the peripheral CD8+ cell and 30% of CD4+ cell repertoire [51], greatly affecting cellular immune responses. Our DEG analyses showed consistent increased gene annotations for herpesviridae infection in frail adults on Days 0, 3, and 7 post-vaccination. In addition to herpesviruses, RNA virus replication was shown to be increased in the frail adults, whereas interferon-associated, anti-viral gene expression was lower compared to the non-frail adults, suggesting reduced immunity against viral infections.

We observed reduced transcripts for ribosomal proteins in frail adults at Day 7 post-vaccination. In contrast, non-frail adults increased ribosomal protein gene expression at Day 7. Ribosome concentration is positively correlated with mRNA translational output [52]. Inhibition of ribosome synthesis was associated with increased CMV infection [53]. Others have found in proteomics analysis, reduced skeletal muscle ribosomal protein levels associated with aging [54]. Our results did not differentiate gene expression by cell types. Further studies using single cell sequencing are needed to provide a clearer picture of how aberrant ribosomal protein expression can affect immune responses to influenza vaccination in the elderly, whether by affecting antibody production, or by affecting *HLA* assembly, expression, antigen processing or presentation.

We also observed concurrent changes in ribosomal protein pseudogenes expression with ribosomal proteins. There have been more than 2000 ribosomal protein pseudogenes described in the literature [55, 56]. The increase in both ribosomal protein and pseudogenes could be due to shared transcription factors, and others have suggested that some ribosomal protein pseudogenes may indeed have protein-coding functions [56, 57].

Mitochondrial dysfunction and reduced mitochondrial content have been suggested as a cause for accelerated frailty [58, 59]. In the frail group, we observed decreased oxidative phosphorylation on Day 7 compared to Day 0, in contrast to the non-frail group in which oxidative phosphorylation gene expression increased from Day 0 to Day 7. mTOR signaling was also reduced in the frail group on Day 7 compared to non-frail group (data not shown). These changes suggest the frail group had reduced energy production/consumption on Day 7 post-vaccination.

Oxidative stress has long been suggested as a mechanism of aging. Increased oxidative stress and altered redox balance have been shown to be associated with frailty [60]. Consistent with these previous studies, our results show that frail adults had increased expression of oxidative pathways, such as heme, *HIF1α*, and glutathione biosynthesis. To our knowledge, our study is the first to link the PBMC transcriptome to frailty, whereas previous studies focused on oxidized plasma proteins/lipids using immunoblots or analytical chemistry methods [61], genomic SNP variations, or epigenetic modification of DNA methylation [62, 63]. Our study provides important insights into oxidative stress pathways in frail adults and may provide therapeutic targets for reducing oxidative stress.

### Strengths and limitations

This was a racially diverse cohort with varying levels of frailty, whose living situation ranged from community-dwelling to long-term care facilities,. We were unable to collect PBMCs from the entire cohort. However, the differences observed between frail and non-frail groups were robust. In the PBMC study, frail and non-frail groups differed by age, with the frail patients being older. Some of the differences found may be related to aging, although corrections for age were made in differential analyses.

Our RNAseq results were only aligned with human genes, thus we have limited information on specific viral infections affecting the subjects’ PBMC populations. It would be interesting to determine if anti-herpes medications for treatments of other types of herpesviruses, such as herpes simplex 1 and 2, and varicella zoster, can affect T cell populations and immunosenescence.

## CONCLUSIONS

This study has identified a number of differences in immune response to influenza vaccination between frail and non-frail older adults. Compared with non-frail older adults, frail adults had reduced expression of genes required for cell proliferation, protein translation, metabolism, antibody production, and interferon expression. The frail group also displayed increased expression of oxidative stress, *IL-8* signaling, and T cell exhaustion genes indicative of immunosenescence. These data shed light on altered transcriptional programs in PBMCs in frail, older adults which are present even in the absence of changes in antibody titers and may contribute to the design of new influenza vaccines. Furthermore, HAI titers may not be the most appropriate assessment of immune response to influenza vaccination among older adults.

## MATERIALS AND METHODS

### Study design and participants

This study was an observational prospective study of immune response to the 2017-2018 influenza vaccine. Participants ≥55 years of age were recruited from primary care practices, the community, and senior living facilities in the fall of 2017, using nonprobability convenience sampling. Eligibility included at least one prior season’s receipt of influenza vaccine, no known allergies to the vaccine or vaccine components, and intention to receive the 2017-2018 influenza vaccine. Participants were ineligible if they had a life expectancy of <6 months, a severe allergy to influenza vaccine or to eggs, a history of Guillain-Barré syndrome, a current immunosuppressive condition or an expected immunosuppressive condition within 6 months, use of immunosuppressant medications, or a history of allograft transplant. The University of Pittsburgh and Centers for Disease Control and Prevention Institutional Review Boards (IRB) approved this study and all participants provided written informed consent prior to initiating study procedures.

### Baseline data collection

Baseline data were collected either by interview, written survey or electronic data retrieval from the electronic medical record (EMR). Demographics included age, sex, race, ethnicity, educational status, smoking status, presence (yes/no) of chronic health conditions, use (yes/no) and name of statin medication. Height and weight were used to calculate Body Mass Index (BMI). BMI was calculated as [weight (lb.) ÷ (height (in.)^2^ X 703]; categorical obesity was defined as BMI ≥30. Functional disability status was assessed using the activities of daily living (ADL) and instrumental activities of daily living (IADL) questionnaires (scores range from 0=low functionality to 14=high functionality). Physical frailty was assessed at baseline using the 5 components outlined in Fried et al.’s Physical Frailty Phenotype [7]. Performance-based components included gait speed (slowness) and hand-grip strength (weakness). Questionnaire-based components included unintentional weight loss ≥10 lbs. in the last year (weight loss), 2-questions from the CES-D Depression scale based on the past week (exhaustion) and 18 activities from the short-version of the Minnesota Leisure Time Activity Questionnaire based on the past 2 weeks. Individual components were either gender-adjusted to specified cut points or adjusted according to specified calculations noted by Fried et al. [7] to determine the frailty score for each component (0=non-frail; 1=frail). Components were then summed (range=0-5). Participants were determined to be frail if they had ≥2 “frail” components and non-frail if they had ≤1 “frail” component.

### Biological samples

Non-fasting whole blood samples were obtained from all participants at Day 0 (pre-vaccination) and 28 (range 19-35) days post influenza vaccination into BD Vacutainer™ serum separator tubes with polymer gel/silica activator additive (BD 367985) for serum HAI determinations and into BD Vacutainer™ CPT™ mononuclear cell preparation tubes with sodium citrate additive (BD 362761) for serum peripheral blood mononuclear cells (PBMCs) on Days 0, 3 (range=3-4) and 7 (range= 6-10) PBMCs were collected for a subgroup of participants who agreed to the additional blood draws and from whom these samples could be obtained. All tubes were held at room temperature and delivered to the processing laboratory within 4 hours of draw. Aliquoted serum samples were frozen at -80° C until assayed.

### Influenza vaccine

Following the Day 0 blood draw, all participants received an intramuscular injection of either the high dose or standard dose seasonal 2017-2018 inactivated influenza vaccine. The trivalent high dose vaccine contained the A/H1N1/Michigan/45/2015-pdm09-like virus, A/H3N2/Hong Kong/4801/2014-like virus and B/Brisbane/60/2008-like influenza virus. The quadrivalent standard dose vaccine also contained the B/Phuket/3073/2013-like virus.

### HAI processing and analysis

Antibody assays were conducted following CDC’s protocols [64] by the Influenza Division research laboratory at CDC, who were blinded to group assignment. Sera were heat inactivated, tested for nonspecific agglutinins, and adsorbed as needed, then serially diluted 2-fold and incubated with 4 hemagglutination units per 25μL of virus with erythrocytes for quantification of HAI titers. Turkey erythrocytes were used for the testing of A/H1N1 viruses. HAI titer was defined as the reciprocal of the last dilution of serum that completely inhibited hemagglutination. Antibody titers <10 (initial sera dilution) were reported as 5 for analysis. Sera were tested in HAI assays against the A/H1N1 vaccine strain included in the 2017-2018 influenza vaccine (A/Michigan/45/2015).

Primary outcome measures were seropositivity at Day 28 and seroconversion of antibodies to influenza A/H1N1. Seropositivity was defined as a HAI titer ≥1:40 at Day 0 and at Day 28. Seroconversion was defined as a 4-fold rise in HAI titer post-vaccination given a pre-vaccination titer ≥10. Secondary outcome measures were log_2_ Day 28 HAI titers and fold-rise in log_2_ HAI titers from Day 0 to Day 28 defined as the ratio of log_2_ Day 28 to log_2_ Day 0 titers.

### PBMC processing and analysis

PBMCs were isolated from serum drawn into CPT™ mononuclear cell preparation tubes with sodium citrate additive (BD 362761) following the manufacturer’s protocol. After isolation, PBMCs were lysed in RLT buffer (Qiagen) and stored at -80° C for RNA isolation using RNeasy kit (Qiagen). Total RNA from PBMCs was isolated from collections at Days 0, 3 and 7 post-vaccination; ribosomal RNA was depleted (Ribo-zero Gold) and sequenced using the Illumina TruSeq platform. Reads per sample were collected and mapped to the Human Ensembl reference genome GRCh38 using the fast alignment tool HISAT2 v2.1.0. EdgeR v3.26.8, an R Bioconductor package, was used for differential gene analysis comparing various sample groups with a gene expression filter of >1 copy per million (CPM) in at least three samples in a given group. Pathway analyses were performed using Ingenuity Pathway Analysis tools (Qiagen). All sequencing data will be provided to GEO Datasets upon publication.

### Statistical analyses

Summary statistics of demographics and immunological response outcomes were conducted for all participants and for the PBMC subgroup by frailty status using Chi-square/Fisher’s exact tests for categorical variables and Wilcoxon/t-tests for continuous variables. Proportions are reported for categorical variables and means and standard deviations or median and quartiles 1 and 3 are reported for continuous variables. Due to the skewness of the HAI titers, they were transformed using the log_2_ function at each timepoint.

The association between frailty and HAI antibody response to influenza vaccine was examined using logistic regression (seroconversion and seropositivity) and linear regression (log_2_ transformed Day 28 antibody titers and fold-rise in log_2_ antibody titers). Analyses were conducted for the entire cohort but not for the PBMC subgroup because sample sizes were inadequate for these analyses. Multivariable regression models included covariates that were significant in univariable analyses (*P* < 0.20) for any outcome measure. Frailty was included *a priori*. Due to the high level of correlation between independent covariates, multivariable stepwise forward regression models were run with *P* ≤ 0.10 to enter and *P* ≤ 0.05 to stay. Statistical significance of two-sided tests was set at type I error (alpha) = 0.05. These analytical procedures were performed using SAS^®^ 9.3 (Cary, NC).

Paired sample analysis (Day 0 to Day 3 or Day 0 to Day 7) was completed using the edgeR function glmFit after normalization of gene counts controlling for age in the model matrix. The false discovery rate (FDR) for each gene was calculated using the Benjamini-Hochberg method because of the multiple null hypotheses being tested. A generalized linear model was used to test for differences in means, correcting for differences in gene expression due to age by performing a likelihood-ratio test with age selected as an adjustment factor [65].

## Supplementary Material

Supplementary Table 1

Supplementary Table 2

Supplementary Table 3

Supplementary Tables 4 and 5

Supplementary Table 6
