# Supplementary material for: The impact of physical frailty on the response to inactivated influenza vaccine in older adults
Source: Aging (Albany NY). 2020 Dec 9;12(24):24633–50. doi: 10.18632/aging.202207 (PMC7803506; doi:10.18632/aging.202207)
Supplement: Supplementary Tables 4 and 5 [file aging-12-202207-s004.pdf]

SUPPLEMENTARY TABLES

Supplementary Table 4. Differentially expressed immunoglobulin genes in frail and non-frail patients.

**Day 0 - 7 Non-frail**		
**Symbol**	**Entrez gene name**	**Expr fold change**
IGHD	immunoglobulin heavy constant delta	-1.235
IGHG1	immunoglobulin heavy constant gamma 1 (G1m marker)	2.06
IGHG3	immunoglobulin heavy constant gamma 3 (G3m marker)	1.3
IGHGP	immunoglobulin heavy constant gamma P (non-functional)	1.51
IGHV1-69	immunoglobulin heavy variable 1-69	-2.128
IGHV4-39	immunoglobulin heavy variable 4-39	1.24
IGKC	immunoglobulin kappa constant	1.28
IGKV1-6	immunoglobulin kappa variable 1-6	1.79
IGKV1-12	immunoglobulin kappa variable 1-12	1.57
IGKV1-16	immunoglobulin kappa variable 1-16	1.45
IGKV1-17	immunoglobulin kappa variable 1-17	1.42
IGKV1-33	immunoglobulin kappa variable 1-33	1.26
IGKV1-39	immunoglobulin kappa variable 1-39 (gene/pseudogene)	1.31
IGKV2-28	immunoglobulin kappa variable 2-28	2.21
IGKV3-20	immunoglobulin kappa variable 3-20	1.43
IGLC2	immunoglobulin lambda constant 2	1.24
IGLC3	immunoglobulin lambda constant 3 (Kern-Oz+ marker)	1.32
IGLV1-40	immunoglobulin lambda variable 1-40	1.41
IGLV1-44	immunoglobulin lambda variable 1-44	1.5
IGLV2-11	immunoglobulin lambda variable 2-11	1.37
IGLV3-21	immunoglobulin lambda variable 3-21	1.7
**Day 0 - 7 Frail**		
**Symbol**	**Entrez gene name**	**Expr fold change**
IGHV1-69D	immunoglobulin heavy variable 1-69D	-1.493
IGKV2-24	immunoglobulin kappa variable 2-24	-3.226
IGKV2D-29	immunoglobulin kappa variable 2D-29	-1.754
IGKV3-15	immunoglobulin kappa variable 3-15	-1.389
IGLL1/IGLL5	immunoglobulin lambda like polypeptide 5	-1.333
IGLV3-19	immunoglobulin lambda variable 3-19	-1.538

Supplementary Table 5. Differentially expressed human leukocyte antigen genes in frail and non-frail patients.

**Day 0 - 7 Non-frail**		
**Symbol**	**Entrez gene name**	**Expr fold change**
HLA-DPA1	major histocompatibility complex, class II, DP alpha 1	1.13
HLA-DPB1	major histocompatibility complex, class II, DP beta 1	1.14
HLA-DQA1	major histocompatibility complex, class II, DQ alpha 1	1.12
HLA-DQB2	major histocompatibility complex, class II, DQ beta 2	1.34
HLA-DRA	major histocompatibility complex, class II, DR alpha	1.23
HLA-DRB1	major histocompatibility complex, class II, DR beta 1	1.16
HLA-DRB5	major histocompatibility complex, class II, DR beta 5	1.18
HLA-G	major histocompatibility complex, class I, G	1.33
HLA-W	major histocompatibility complex, class I, W (pseudogene)	-1.235
**Day 0 - 7 Frail**		
**Symbol**	**Entrez gene name**	**Expr fold change**
HLA-DRA	major histocompatibility complex, class II, DR alpha	-1.176
HLA-G	major histocompatibility complex, class I, G	-1.389
HLA-J	major histocompatibility complex, class I, J (pseudogene)	-1.299
